# Light gradients and optical microniches in coral tissues

**DOI:** 10.3389/fmicb.2012.00316

**Published:** 2012-08-27

**Authors:** Daniel Wangpraseurt, Anthony W. D. Larkum, Peter J. Ralph, Michael Kühl

**Affiliations:** ^1^Plant Functional Biology and Climate Change Cluster, Department of Environmental Sciences, University of Technology SydneySydney, NSW, Australia; ^2^Marine Biological Section, Department of Biology, University of CopenhagenHelsingør, Denmark; ^3^Singapore Centre on Environmental Life Sciences Engineering, School of Biological Sciences, Nanyang Technological UniversitySingapore, Singapore

**Keywords:** coral photobiology, bio-optics, microenvironment, tissue optics, zooxanthellae, microsensor, microgradients, ecophysiology

## Abstract

Light quantity and quality are among the most important factors determining the physiology and stress response of zooxanthellate corals. Yet, almost nothing is known about the light field that *Symbiodinium* experiences within their coral host, and the basic optical properties of coral tissue are unknown. We used scalar irradiance microprobes to characterize vertical and lateral light gradients within and across tissues of several coral species. Our results revealed the presence of steep light gradients with photosynthetically available radiation decreasing by about one order of magnitude from the tissue surface to the coral skeleton. Surface scalar irradiance was consistently higher over polyp tissue than over coenosarc tissue in faviid corals. Coral bleaching increased surface scalar irradiance by ~150% (between 500 and 700 nm) relative to a healthy coral. Photosynthesis peaked around 300 μm within the tissue, which corresponded to a zone exhibiting strongest depletion of scalar irradiance. Deeper coral tissue layers, e.g., ~1000 μm into aboral polyp tissues, harbor optical microniches, where only ~10% of the incident irradiance remains. We conclude that the optical microenvironment of corals exhibits strong lateral and vertical gradients of scalar irradiance, which are affected by both tissue and skeleton optical properties. Our results imply that zooxanthellae populations inhabit a strongly heterogeneous light environment and highlight the presence of different optical microniches in corals; an important finding for understanding the photobiology, stress response, as well as the phenotypic and genotypic plasticity of coral symbionts.

## INTRODUCTION

Coral reefs are among the most productive and diverse ecosystems on Earth and their evolutionary success can be largely attributed to the successful interaction between scleractinian corals and their associated microorganisms, most importantly their microalgal photosymbionts (zooxanthellae) belonging to the dinoflagellate genus *Symbiodinium*. The quantity of light is a key environmental parameter regulating the nature of this photosymbiosis (Falkowski et al., 1990). Under optimal irradiance regimes, light stimulates symbiont photosynthesis, which provides organic carbon for the coral animal that in turn provides metabolic waste products supporting zooxanthellae photosynthesis (Muscatine et al., 1981). Excess quantities of light, however, readily lead to photoinhibition and can damage the photosynthetic apparatus. Light in combination with elevated temperature can lead to the expulsion of the zooxanthellae (and/or pigment degradation) and the breakdown of the symbiosis (Lesser, 1996; Jones et al., 1998; Warner et al., 1999). This breakdown, termed coral bleaching, has been intensively studied over the last decades, including a primary focus on the photobiology of zooxanthellae (Glynn, 1996; Brown, 1997; Hoegh-Guldberg, 1999). Despite such efforts, it is surprising that virtually nothing is known about the actual light regime surrounding the zooxanthellae *in hospite*, i.e., within the coral tissue, albeit the light microenvironment is a central control factor of the photo- and stress physiology of zooxanthellae and their coral hosts.

The optical environment within the host tissue is likely to vary substantially in relation to the ambient macro-environment. First, direct micro-scale measurements of photon scalar irradiance (i.e., the integral quantum flux incident from all directions about a given point) on the coral tissue surface revealed scalar irradiance values reaching up to 200% of the incident downwelling photon irradiance (Kühl et al., 1995). Such enhancement is currently thought to mainly result from multiple scattering of photons in the coral skeleton below the tissue (Enriquez et al., 2005). The aragonite skeleton scatters light isotropically so that photons interacting with the skeleton are diffusely backscattered into the tissue (Enriquez et al., 2005). Diffuse scattering increases the path length of photons per vertical distance traversed, i.e., it enhances the average residence time of photons at a given depth horizon and can thereby lead to local enhancement of scalar irradiance (Kühl and Jørgensen, 1994; Enriquez et al., 2005). It is currently assumed that the light field within the coral tissue is diffuse and uniformly enhanced over the incident irradiance (Enriquez et al., 2005; Teran et al., 2010). However, the optical environment within the coral may be more complex as tissue–light interactions and the optical properties of coral tissue remain largely unexplored.

Photons interacting with tissue can have three different fates: (i) simple unimpeded transmission; (ii) absorption followed by either red-shifted re-emission (as fluorescence or phosphorescence), heat dissipation or dissipation via photochemical reactions such as photosynthesis or radical formation; (iii) scattering and diffraction leading to a redirection of photons out of their original path. The occurrence of these events is determined by a complex interplay between the nature and direction of incident light and the optical properties of the given tissue (Wilson and Jacques, 1990). The optical properties of living tissue are best studied for human skin, but also well-described for terrestrial plants (Anderson and Parrish, 1981; Vogelmann, 1993) as well as aquatic sediments and biofilms (Kühl and Jørgensen, 1994). The development and use of fiber-optic microprobes (Vogelmann et al., 1991; Kühl, 2005) has facilitated experimental investigation of light microenvironments and optical properties within such systems (Vogelmann and Björn, 1984; Vogelmann, 1993; Vogelmann et al., 1996). Besides a few preliminary measurements (Kühl et al., 1995; Kaniewska et al., 2011), comparable studies on coral tissue are lacking. Kaniewska et al. (2011) mainly focused on comparing larger scale heterogeneity of light fields in different corals and presented only few spot measurements of scalar irradiance at a fixed depth in the coral tissue and no detailed vertical or lateral profiling was done. The presence and nature of micro-scale heterogeneity in coral light fields, both laterally over different coral tissue types and vertically within a given tissue type, have thus not been resolved.

Here we used scalar irradiance microprobes (Vogelmann and Björn, 1984; Lassen et al., 1992) to characterize the spectral light field and light penetration in coral tissues. The specific aims were (1) to directly measure light penetration in tissue of corals belonging to the family Faviidae, (2) investigate the effect of tissue type (coenosarc and polyp tissue) and loss of pigmentation (bleaching) on light microenvironments for a variety of abundant coral species, and (3) investigate how gradients of light and photosynthesis within coral tissue align with each other. Our results provide the first insight into the basic optical properties of coral tissue and describe the *in hospite* optical microenvironment of corals from a zooxanthellar perspective.

## RESULTS

### LIGHT FIELDS SURROUNDING CORAL TISSUE

**FIGURE 1:**
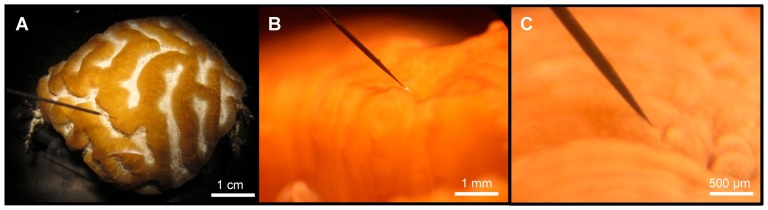
**Micro-scale scalar irradiance measurements on corals. (A)** Overview showing a small fragment of *Platygyra lamellina* with the scalar irradiance microprobe positioned at the coral tissue surface at a 45° angle, **(B)** the spherical microsensor tip (white bulb; 100 μm) at the surface of coenosarc tissue, and **(C)** the microprobe inserted into coral tissue.

**FIGURE 2:**
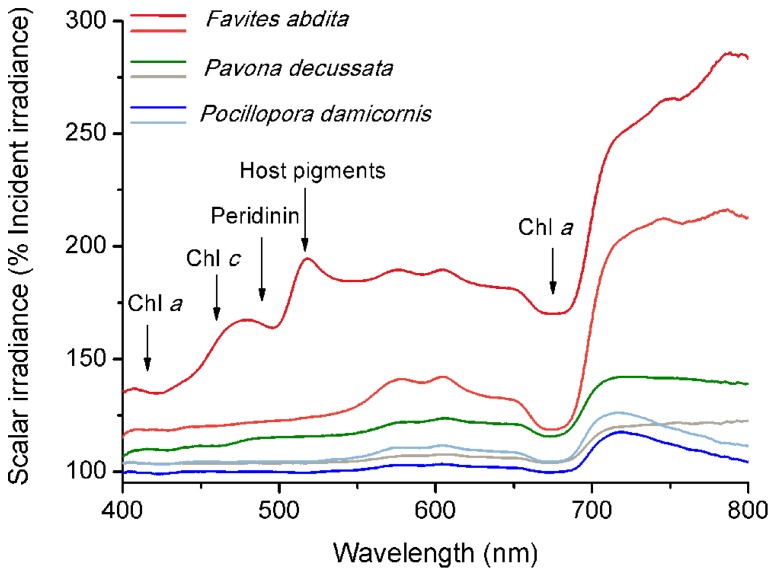
**Micro-scale spectral scalar irradiance at the tissue surface of different corals.** Data were normalized to the incident downwelling spectral irradiance, *E*_d_. Note that scale begins at 100% *E*_d_. Dark and light color tones represent measurements made on polyp and coenosarc tissue, respectively. Arrows show major absorption wavelengths of peridinin (480–490 nm), chlorophyll *c* (460 nm) and chlorophyll *a* (435–440, 675 nm), and emission/reflectance of host pigments (480–590 nm).

**FIGURE 3:**
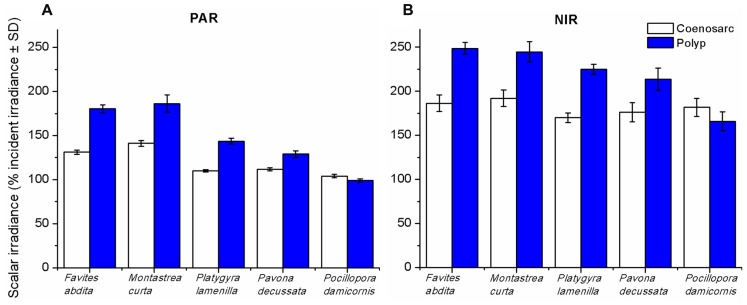
**Integrated scalar irradiance (in % of downwelling irradiance) at the tissue surface of corals.** **(A)** PAR (photosynthetically available radiation, 400–700 nm) and **(B)** NIR (near-infrared radiation, 700–800 nm). Data are means ± SD (*n* = 9). Measurements were done at the surface of the coenosarc (white bars) and polyp (blue bars) tissue, respectively.

**FIGURE 4:**
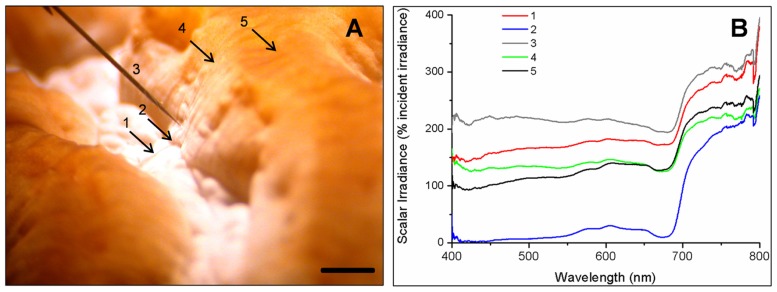
**Mapping of spectral scalar irradiance heterogeneity along one coral polyp in *Platygyra lamellina*.** **(A)** Photograph detailing the five different measurement positions along a horizontal tissue surface gradient (indicated as arrow or sensor tip, 1–5). Black scale bar is 2 mm. **(B)** Single scalar irradiance spectra (400–800 nm) at the measurement points (1–5).

Spectral scalar irradiance at the coral tissue surface (**Figure 1**) differed markedly between tissue types (i.e., coenosarc and polyp) both within a coral species and between species, despite identical regimes of incident collimated irradiance (**Figure 2**). Generally, there was an enhancement in scalar irradiance over incident irradiance reaching maximum values of ~180% for photosynthetically active radiation (PAR, 400–700 nm) and ~250% for near-infrared radiation (NIR, 700–800 nm). Scalar irradiance levels in faviid corals were systematically higher over polyp tissue than over coenosarc tissue for both PAR and NIR (**Figures 2 and 3A,B**). For example, the scalar irradiance measured over polyp tissue of *Favites abdita*, was about 1.4 and 1.3 times higher than over coenosarc tissue for PAR and NIR, respectively (**Figures 3A,B**). In contrast, no differences between polyp and coenosarc tissue were present in *Pocillopora damicornis* (**Figures 3A,B**). Characteristic absorption peaks of Chl *a* (430–440, 675 nm), Chl *c* (460 nm), and the carotenoid peridinin (480–490 nm) were found at the surface of faviid corals; these peaks were especially pronounced over polyp tissue. In comparison, spectra of *Pavona decussata* and *Pocillopora damicornis* showed less distinct spectral signatures (**Figure 2**). More detailed horizontal mapping of scalar irradiance across the tissue surface (from polyp mouth over walls to coenosarc) revealed strong small-scale heterogeneity (**Figure 4**), but consistent results were obtained between the two different tissue types (coenosarc and polyp mouth tissue). Pigmentation across the polyp walls appeared heterogeneous (patchy distribution of host pigments) as were the measurements of scalar irradiance. When the sensor was placed within a tissue fold (i.e., sphere entirely covered by tissue), irradiance was significantly attenuated (**Figure 4**).

### EFFECTS OF CORAL BLEACHING ON TISSUE SURFACE SPECTRAL SCALAR IRRADIANCE

**FIGURE 5:**
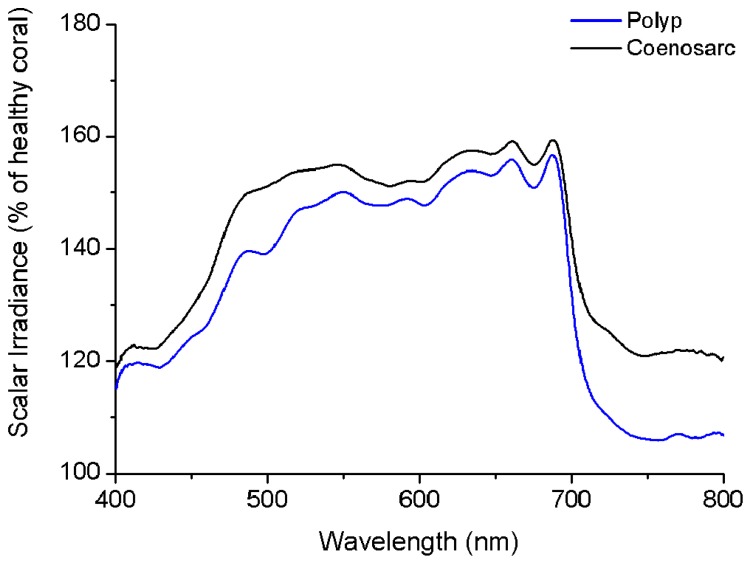
**Effect of bleaching on the tissue surface scalar irradiance of a coral.** The figure shows spectral scalar irradiance for coenosarc (black) and polyp (blue) tissue of a bleached *Goniastrea aspera*, expressed as percentage of healthy coral.

Loss of pigmentation (bleaching) led to a further increase in the tissue surface scalar irradiance of corals reaching ~150% of the scalar irradiance levels of a healthy coral at wavelengths between ~500 and 700 nm (**Figure 5**). However, in the blue region of visible light (~400–500 nm) and in the NIR, bleaching only led to a slight (10–20%) increase in scalar irradiance. Bleaching affected the light field of coenosarc tissue to a greater extent than that of polyp tissue with greatest deviations of >10% occurring between ~470–510 and 720–800 nm.

### SPECTRAL SCALAR IRRADIANCE WITHIN CORAL TISSUE

**FIGURE 6:**
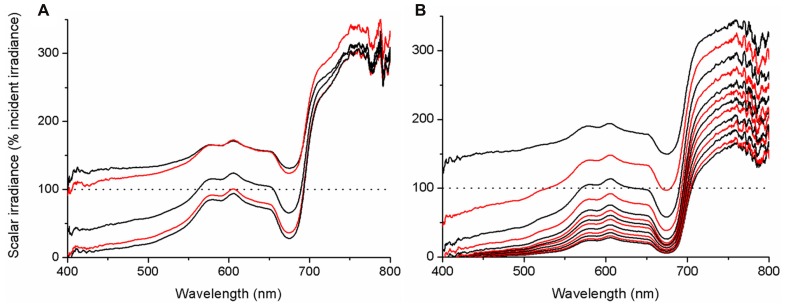
**Microprofiles of spectral scalar irradiance within coral tissue.** Representative profiles measured in the coral *Montastrea curta* within **(A)** coenosarc (tissue thickness ~400 μm) and **(B)** polyp tissue (tissue thickness ~1200 μm). Scalar irradiance was normalized to the incident downwelling spectral irradiance, *E*_d_; dotted line represents 100% *E*_d_. The uppermost spectrum (black) represents measurements taken at the coral surface and subsequent spectra correspond to increments of 100 μm with the lowermost spectrum equaling measurements over the coral skeleton. Spectra are colored in an alternating fashion (black–red) for clarity.

**FIGURE 7:**
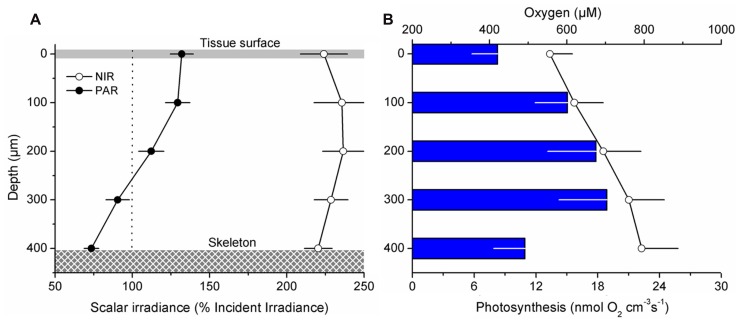
**Microgradients of light, O_2_, and photosynthesis within coral tissue.** Microprofiles were measured within ~400 μm thick coenosarc tissues of the coral *Montastrea curta* from just above the skeleton (depth 400 μm) to the coral surface (depth 0 μm). Data points are mean values (±SE; *n* = 4) **(A)** Mean photosynthetically available radiation (PAR, 400–700 nm; closed symbols) and near-infrared radiation (NIR, 700–800 nm; open symbols) scalar irradiance profiles. Scalar irradiance was normalized to the incident downwelling irradiance. **(B)** Average O_2_ concentration (μM; open symbols) and gross photosynthesis (nmol O_2_ cm^-3^ s^-1^; blue bars). For clarity error bars of O_2_ concentration and photosynthesis are only + and -, respectively. The downwelling photon irradiance *E*_d_ (PAR) was 640 μmol photons m^-2^ s^-1^.

Our microprofiles revealed the presence of strong light gradients within coral tissue (**Figures 6A,B**). Over the visible range (PAR, 400–700 nm), scalar irradiance was attenuated toward the skeleton. For coenosarc tissue, scalar irradiance of PAR decreased from ~132 to 74% of the incident downwelling irradiance over a distance of 400 μm. Such decrease in PAR was even more pronounced for a 1100-μm thick polyp tissue, where PAR scalar irradiance decreased from ~176 to 16% of the incident downwelling irradiance. In many cases, we noted a subsurface maximum ~50–100 μm below the tissue surface, where no attenuation or even an increase in scalar irradiance occurred. This subsurface maximum was most pronounced at wavelengths around 550–650 nm (**Figure 6A**), i.e., outside major absorption peaks of photopigments. Most of the light was absorbed in the upper tissue layers and spectra close to the skeleton were more similar in intensity (**Figures 6A,B**). In the NIR region, no attenuation occurred within coenosarc tissue and high values of around 220–240% of incident downwelling irradiance remained (**Figures 6A and 7A**). In contrast, NIR did significantly attenuate from surface to skeleton inside polyp tissue from ~306 to 124% of the incident downwelling irradiance (**Figure 6B**).

### OXYGEN MICROENVIRONMENT AND PHOTOSYNTHESIS WITHIN CORAL TISSUE

Oxygen measurements within illuminated coral tissue revealed a steady increase in O_2_ concentration from the tissue surface toward the skeleton (**Figure 7B**). Maximum O_2_ concentrations at the tissue–skeleton interface reached ~800 μM (~400% air saturation), which was about 240 μM higher than the O_2_ concentration at the tissue surface. Gross photosynthesis increased with distance from the surface until ~300 μm within the tissue, where it peaked at ~18.9 (±4.7 SE) nmol O_2_ cm^-3^ s^-1^. The peak in gross photosynthesis correlated with a zone in the tissue exhibiting the highest scalar irradiance attenuation (compare **Figures 7A,B**). Photosynthesis was lowest at the coral tissue surface and at the tissue–skeleton interface (8.3 ± 2.5 and 10.9 ± 3.0 nmol O_2_ cm^-3^ s^-1^, respectively; **Figure 7B**).

**FIGURE 8:**
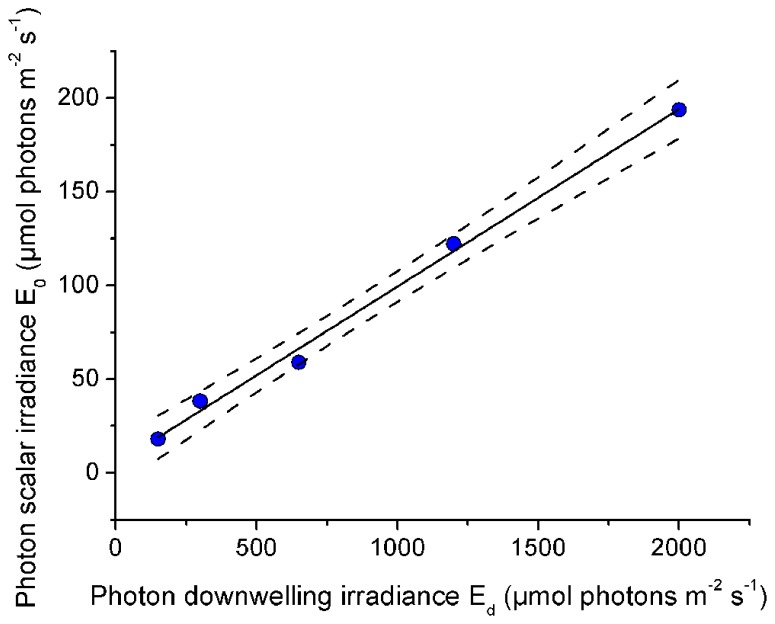
**Effect of incident photon irradiance on within tissue photon scalar irradiance (400–700 nm).** The microsensor was positioned at a depth of 1000 μm within the polyp tissue just above the skeleton of the coral *Platygyra lamellina*. Black line illustrates a linear fit (*r*^2^ > 0.99) and dashed lines represent ±95% confidence intervals.

The relationship between incident downwelling photon irradiance and the photon scalar irradiance for visible wavelengths (400–700 nm) was measured at the tissue–skeleton interface ~1000 μm within polyp tissue. There was a constant linear relationship between incident irradiance and the scalar irradiance level at the tissue–skeleton interface (*r*^2^ > 0.99; **Figure 8**). Over the range of experimental downwelling photon irradiances (150–2000 μmol photons m^-2^ s^-1^) only about 1/10 was measured as photon scalar irradiance within this optical niche.

## DISCUSSION

In this study, we used fiber-optic microprobes to obtain the first detailed measurements of vertical and lateral light gradients within and across coral tissues in several species. While the chemical microenvironment of corals has been explored in several studies since microsensors were introduced to coral research (Kühl et al., 1995), only a few examples of scalar irradiance measurements in corals have been published and these have been hampered by difficulties in entering and/or precise positioning in the tissue (Kühl et al., 1995; Kaniewska et al., 2011). Hitherto, tissue effects on coral light fields have largely been ignored in coral optics studies that have mostly focused on the role of diffuse backscatter from the coral skeleton and from the coral tissue into the surrounding seawater (Enriquez et al., 2005; Teran et al., 2010). Combining micro-incision with scalar irradiance profiling, we have now unequivocally demonstrated the presence of light gradients in corals and present the first evidence that tissue optics is an important factor to consider in coral photobiology.

Direct *in hospite* micro-scale light measurements in corals differ from predictions in previous modeling studies, which have calculated that internal irradiance is homogenously enhanced compared to the external environment based on known downwelling irradiance regimes (Enriquez et al., 2005; Teran et al., 2010). We show that a clear spatial stratification exists within coral tissue, where scalar irradiance in the upper coral tissue layers (0–100 μm) can reach up to 200% of incident downwelling irradiance, whilst lower cell layers are subject to more light-limiting conditions (**Figures 6B and 8**). Our results thus suggest that the light microhabitat of corals is not only determined by the properties of the skeleton, but also largely by the characteristics of the tissue.

Until now, coral tissue has simply been treated as a thin layer of light absorbing particles (i.e., *zooxanthellae*) on top of the light-diffusing skeleton and overlain by seawater (Teran et al., 2010). We know from other systems, however, such as plant leafs or animal skin, that the properties of the tissue itself can significantly enhance light fields at the tissue interface due to scattering and internal reflection (Anderson and Parrish, 1981; Vogelmann et al., 1996; Spilling et al., 2010). The peak of scalar irradiance observed here in the upper cell layers suggests that substantial scattering and photon-trapping must occur at the tissue–water interface, potentially resulting from a mismatch in the refractive index of coral tissue and water (Kühl and Jørgensen, 1994). Nevertheless, our results also confirm that the earlier-reported diffuse scattering component of the skeleton is functional *in hospite* (Enriquez et al., 2005), as seen by a decrease in light attenuation toward the skeleton surface (**Figure 6B**). The occurrence and significance of skeleton backscatter is further exemplified by the continuous enhancement of NIR throughout the coenosarc tissue, where no pigments are present that absorb over these wavelengths (**Figure 7A**).

We hypothesized that with increasing incident irradiance, within-tissue PAR would increase exponentially as more light would be transmitted through the tissue and interact with the skeleton, thereby increasing the relative importance of backscattered light from the skeleton at the tissue–skeleton interface. However, we found a constant linear relationship between PAR at the tissue–skeleton interface and incident PAR (**Figure 8**). Photons are thus efficiently absorbed before they get scattered by the skeleton, indicating that the coral tissue itself also contributes to the high efficiency of light absorption found in corals (Stambler and Dubinsky, 2005). Scalar irradiance at the tissue surface increased by 150% in a bleached coral relative to the surface scalar irradiance in a healthy coral (**Figure 5**). This was less than expected according to coral skeleton scattering theory (Teran et al., 2010) and again suggests that other light redistributing mechanisms occur within the tissue. However, the nature of light gradients and thus the relative importance of tissue vs skeleton optics will be variable. Coral tissue varies in thickness, metabolite composition, symbiont and host pigment distribution, and abundance, all of which modulate coral tissue optics. Additionally, the role of skeleton optics is variable due to differences in morphology and density. For instance, thick corallite walls guide more light into the coral interior, whilst more dense structures facilitate diffuse backscattering (Highsmith, 1981). Therefore, the optical microenvironment within corals is the result of a complex interplay between skeleton and tissue optical properties, which clearly deserves further investigations.

Coral tissue surface scalar irradiance differed on a spatial scale between coral species and tissue types, despite identical incident irradiance regimes (**Figures 3A,B**). Since we excluded the potential for any interference with colony and/or macro-scale light-regulating factors such as colony morphology and orientation (Anthony et al., 2005), we conclude that the observed differences are caused by micro-scale optical properties of coral tissue and skeleton. In faviid corals, host pigments are often locally concentrated toward the polyp mouth (e.g., Salih et al., 2000; Oswald et al., 2007; but see spectral signatures in **Figure 2**). The enhanced tissue surface scalar irradiance of polyp over coenosarc tissue may be explained partly by the presence of such pigments, which effectively reflect, fluoresce, and scatter light (Schlichter et al., 1988; Salih et al., 2000).

Previous studies have shown the presence of tissue type-related spatial heterogeneity in photosynthesis (Ralph et al., 2002; Hill et al., 2004; Al-Horani et al., 2005). For instance, in the coral *Galaxea fascicularis* O_2_ production was shown to be about 10 times higher over polyp than over coenosarc tissue (Al-Horani et al., 2005). Such differences may likely be related to distinct light microenvironments in the coral tissue. For productivity comparisons between species under identical incident irradiance regimes it appears crucial to consider the ability of corals to modulate their own light regime by skeleton structure and tissue organization/movement (**Figures 3A,B**). Tissue and skeleton optical properties have a strong effect on the local light environment that may partly explain observed species- and tissue type-related differences in photosynthesis.

Our results show that *Symbiodinium* populations, inhabiting oral and aboral coral tissue layers of faviid corals, experience steep light gradients with scalar irradiance reaching down to 10% of the surface irradiance in deeper tissue layers (**Figure 6B**); the vertical attenuation of light observed in the coral tissue over a few hundred microns is comparable to the reduction in irradiance that occurs between surface waters and >25 m depth in oceanic waters (Kirk, 1994). Our findings thus call for a revision of the current view on the optical environment surrounding zooxanthellae.

On the scale of a single colony, irradiance gradients between light exposed and shaded tissue can lead to both a distinct distribution of *Symbiodinium* clades and/or differential photoacclimation of the latter (Rowan et al., 1997; Toller et al., 2001; Ulstrup et al., 2006; Sampayo et al., 2007). The potential for such mechanisms occurring within tissue on a vertical micro-scale, for instance between oral and aboral tissue layers, is not known for corals, but is well-studied for terrestrial leaves (Schreiber et al., 1996). It is, e.g., known that shade-adapted chloroplasts exist in the lower tissue layers of sun-adapted leaves and chloroplasts deep within leaves are photoacclimated to local irradiance regimes (Terashima, 1989). We found that maximum rates of photosynthesis occurred in lower parts of coral tissue and not at the surface where scalar irradiance was at its maximum (**Figure 7B**). In fact, the spatial relationship between photosynthesis and light observed here is similar to results obtained from spinach leaves where photosynthetic O_2_ production showed a peak deep within the leaf, whilst irradiance maxima were obtained at the top part of the leaf (Nishio et al., 1993). These findings underscore the potential for photoacclimation to different light microclimates within coral tissue.

Clades and sub-clades of *Symbiodinium* exhibit a range of light-harvesting strategies (Reynolds et al., 2008; Ragni et al., 2010; Kraemer et al., 2012) and it will be interesting in the future to ascertain the location of various clades in coral species that harbor more than one clade, relative to the actual light field characteristics. The optical environment is a primary factor controlling the activity and distribution of phototrophic organisms and the presence of intratissue light gradients must have an effect on the ecophysiology of zooxanthellae in yet unknown ways.

Our results also have implications for the understanding of coral bleaching patterns. It has been observed that thick-tissued corals survive stress events better than thin-tissued ones (Loya et al., 2001). It has also been hypothesized that thick coral tissue could provide sheltered light environments for resident zooxanthellae, thereby increasing stress resilience and the survival of thick-tissued corals (Hoegh-Guldberg, 1999). We show here that thick-tissued corals do indeed harbor such sheltered optical microniches (**Figures 6A,B**). This photoprotection is substantial as even under conditions of stressful excess radiation (incident PAR irradiance levels of ~2000 μmol photons m^-2^ s^-1^) thick coral tissue can harbor low light niches for photosynthesis experiencing about 1/10 of incident irradiance (200 μmol photons m^-2^ s^-1^; **Figure 8**).

Yet another option is that habitat heterogeneity is favored in thick-tissued corals, which in turn leads to a larger symbiont pool with diverse phenotypic or genotypic characteristics and stress resilience (Rowan et al., 1997). We found habitat heterogeneity both in the optical and the chemical environment (**Figures 7A,B**). The coral skeleton represents a diffusion barrier for chemical species, which will lead to a relative build-up of gases in lower tissue layers toward the tissue–skeleton interface as shown here by an increasing O_2_ concentration up to ~400% air saturation (**Figure 7B**). Thus, tissue thickness will favor microenvironmental heterogeneity. Whether this then favors a greater pool of symbiont populations (or subpopulations) and if this translates to increased stress resilience remains to be investigated.

In conclusion, we show here the first evidence for the presence of strong light gradients within the tissue of symbiotic corals. The optical properties of coral tissue have an important role in controlling microenvironmental light fields within corals. Our results imply that zooxanthellae within one single polyp can be subject to different light microenvironments with irradiance levels spanning over one order of magnitude. These results call for a revision of our current understanding of the interaction between light and corals and provide the very basis for future investigations on microenvironmental optical controls of coral photo- and stress physiology.

## MATERIALS AND METHODS

### CORAL SAMPLES

Corals were collected from shallow waters (<3 m depths) on the reef flat of the Heron Island lagoon, Great Barrier Reef, Australia (152°06′E, 20°29′S). We selected several species of faviid corals (*Favites abdita*, *Goniastrea aspera*, *Montastrea curta*, *Platygyra lamellina*) suitable for intratissue microsensor measurements (i.e., thick tissue and minimal mucus secretion; Alieva et al., 2008). We also sampled small coral fragments (<5 cm diameter) of the branching *Pocillopora damicornis* (Pocilloporidae) and the foliaceous *Pavona decussata* (Agariciidae) because they have contrasting micro-scale properties (pigment composition and microtopography) compared to faviids. Samples were transported to the permanent coral holding facility at the University of Technology, Sydney, where corals were maintained under continuous flow at 25°C, salinity of 33 and a photon irradiance of 200 μmol photons m^-2^ s^-1^ (400–700 nm; 12/12 h light–dark cycle).

### SCALAR IRRADIANCE MICROSENSOR MEASUREMENTS

Experiments were conducted with coral fragments placed in a custom-made black acrylic flow chamber supplied with seawater (as above) at a flow velocity of ~3 cm s^-1^. Samples were illuminated vertically from above with a collimated light beam from a fiber-optic tungsten-halogen lamp (KL-2500, Schott GmbH, Germany), equipped with a heat filter and a collimating lens. The complete set-up was covered with black cloth to avoid stray light. Fiber-optic scalar irradiance microprobes with a spherical tip diameter of 80–100 μm were used to map light microenvironments in the corals (Lassen et al., 1992). The microprobes were mounted on a PC-controlled motorized micromanipulator for automatic profiling (Pyro-Science GmbH, Germany), at an angle of 45° relative to the vertically incident light (to avoid self-shading, see Lassen et al., 1992). The micromanipulator was fixed onto a heavy-duty vibration-free metal stand. Scalar irradiance spectra were recorded with the microprobes connected to a PC-controlled fiber-optic spectrometer controlled by the manufacturers software (USB2000+ and *Spectrasuite*, Ocean Optics, USA). Positioning of the microprobe was facilitated by the manufacturers software (Profix, Pyro-Science GmbH, Germany).

Scalar irradiance at the coral tissue surface was mapped at coenosarc (tissue connecting two polyps) and polyp tissue for the investigated coral species (**Figure 1**). These tissue types were chosen because of known differences in photobiology and pigment composition (e.g., Hill et al., 2004; Ulstrup et al., 2006). First, reference measurements of the incident downwelling irradiance (*E*_d_) were done over a black non-reflective surface as described previously (e.g., Kühl and Jørgensen, 1994), followed by measurements at the coral surface. The coral surface was defined as the depth where the sphere of the sensor just touched the tissue. By re-arranging the aquarium and/or the coral, but not the sensor or the incident light, we were able to measure coral surface irradiance at the identical spot in the incident light field, where *E*_d_ was measured, thereby avoiding potential artifacts due to heterogeneities in the incident light field. Differences in scalar irradiance were thus solely related to the optical properties of corals. Since we were interested in understanding how coral micro-scale properties and not gross morphology (i.e., growth form, etc.) modulate tissue light regimes, we only measured in locations that were directly exposed to the vertically incident collimated light beam. For each of the measured coral species, nine replicate measurements were done on randomly chosen coral polyps.

To investigate the impact of reduced pigmentation on coral tissue optics, we also measured a bleached and normally (dark-brownish) pigmented piece of *Goniastrea aspera*. The bleached coral originated from an experimental coral tank and was assessed to be severely bleached according to the color reference chart (score D1 = Chl *a* density: <1 μg cm^-2^, cells: <1 × 10^5^ cm^-2^; see Siebeck et al., 2006). The tissue structure was visually assessed as healthy and uncompromised.

For measuring light gradients inside the coral tissue, a micro-incision had to be made in order to allow smooth tissue penetration by the sensor without indentation – a major problem in earlier attempts to measure light profiles in coral tissue. We found that a double-edged diamond knife (60°, blade thickness 200 μm, effective blade thickness <100 μm; ProSciTech Pty Ltd, USA) was very suitable for such purpose. Diamond knifes are commonly used in tissue micro-surgery because they perform highly precise and minimally invasive cuts. Incisions were made carefully under a dissecting microscope with the knife inserting the animal tissue at diagonal angles until the skeleton was reached. After the incision, the coral tissue contracted somewhat and after ~5–10 min extrusion of mesenterial filaments, and tissue movement, often started coating sensor tips and interfering with the measurements. Therefore, measurement of each scalar irradiance profile commenced immediately after the incision was made and was finished within ~1–3 min. For each profile, the microsensor was carefully inserted into the cut until the skeleton surface was reached, by means of the micromanipulator and the automatic profiling function of the motor (step sizes of 50–100 μm). The position of the skeleton surface within the tissue was easily identified as a minimal retraction/bending of the optical fiber. Subsequently, profiling was done upward from the skeleton surface into the overlying tissue in steps of 100 μm logging the average of 10 spectra at each measuring depth until reaching the tissue surface (**Figure 1**).

To investigate the effect of changes in the ambient irradiance regime on the within tissue scalar irradiance we measured scalar irradiance within the polyp tissue (~1000 μm depth) of *Platygyra lamellina* under increasing levels of downwelling photon irradiance spanning the range from 0 to 2000 μmol photons m^-2^ s^-1^.

Raw spectra were integrated between (400–700 nm, PAR) and (700–800 nm, NIR) using the mathematical integration function of Origin Pro 8.0 (Origin, USA). Spectral profiles were normalized to *E*_d_ and smoothed if noisy, using the Savitzky–Golay function of Origin with a 50 point window; visual inspection of spectra before and after smoothing showed no loss of spectral details.

### O_2_ MICROSENSOR MEASUREMENTS

We used Clark-type O_2_ microelectrodes with a tip size of 25 μm, a 90% response time of <0.5 s and a stirring sensitivity of ~1% (Revsbech, 1989; OX25, Unisense AS, Denmark). Sensors were linearly calibrated against air saturated water and anoxic water (flushed with N_2_). The percent air saturation in the seawater at experimental temperature and salinity was transformed to O_2_ concentration (μmol O_2_ L^-1^) as described previously (Garcia and Gordon, 1992). The O_2_ microsensor measurements inside the coral tissue were conducted using a similar approach as with the fiber-optic microprobes (see above). However, the O_2_ microsensors approached the coral surface at an angle of ~10° relative to the vertical as this facilitated better tissue penetration. For each measuring depth, we estimated steady state O_2_ concentrations followed by gross photosynthesis estimates using the light–dark shift technique (see Revsbech and Jørgensen, 1983 for detailed description). Data were recorded with a conventional strip-chart recorder with a rapid time response (BD25, Kipp & Zonen, The Netherlands).

## AUTHOR CONTRIBUTIONS

Daniel Wangpraseurt and Michael Kühl designed research; Daniel Wangpraseurt performed research; Anthony W. D. Larkum, Peter J. Ralph, and Michael Kühl contributed new reagents/analytic tools; Daniel Wangpraseurt, Anthony W. D. Larkum, and Michael Kühl analyzed data; and Daniel Wangpraseurt, Anthony W. D. Larkum, and Michael Kühl wrote the paper.

## Conflict of Interest Statement

The authors declare that the research was conducted in the absence of any commercial or financial relationships that could be construed as a potential conflict of interest.
